# Corticospinal Tract Wiring and Brain Lesion Characteristics in Unilateral Cerebral Palsy: Determinants of Upper Limb Motor and Sensory Function

**DOI:** 10.1155/2018/2671613

**Published:** 2018-09-13

**Authors:** Cristina Simon-Martinez, Ellen Jaspers, Lisa Mailleux, Els Ortibus, Katrijn Klingels, Nicole Wenderoth, Hilde Feys

**Affiliations:** ^1^Department of Rehabilitation Sciences, KU Leuven-University of Leuven, Leuven, Belgium; ^2^Neural Control of Movement Lab, Department of Health Sciences and Technology, ETH Zurich, Zurich, Switzerland; ^3^Department of Development and Regeneration, KU Leuven-University of Leuven, Leuven, Belgium; ^4^Rehabilitation Research Centre, BIOMED, Hasselt University, Diepenbeek, Belgium

## Abstract

Brain lesion characteristics (timing, location, and extent) and the type of corticospinal tract (CST) wiring have been proposed as determinants of upper limb (UL) motor function in unilateral cerebral palsy (uCP), yet an investigation of the relative combined impact of these factors on both motor and sensory functions is still lacking. Here, we first investigated whether structural brain lesion characteristics could predict the underlying CST wiring and we explored the role of CST wiring and brain lesion characteristics to predict UL motor and sensory functions in uCP. Fifty-two participants with uCP (mean age (SD): 11 y and 3 m (3 y and 10 m)) underwent a single-pulse Transcranial Magnetic Stimulation session to determine CST wiring between the motor cortex and the more affected hand (*n* = 17 contralateral, *n* = 19 ipsilateral, and *n* = 16 bilateral) and an MRI to determine lesion timing (*n* = 34 periventricular (PV) lesion, *n* = 18 corticosubcortical (CSC) lesion), location, and extent. Lesion location and extent were evaluated with a semiquantitative scale. A standardized protocol included UL motor (grip strength, unimanual capacity, and bimanual performance) and sensory measures. A combination of lesion locations (damage to the PLIC and frontal lobe) significantly contributed to differentiate between the CST wiring groups, reclassifying the participants in their original group with 57% of accuracy. Motor and sensory functions were influenced by each of the investigated neurological factors. However, multiple regression analyses showed that motor function was predicted by the CST wiring (more preserved in individuals with contralateral CST (*p* < 0.01)), lesion extent, and damage to the basal ganglia and thalamus. Sensory function was predicted by the combination of a large and later lesion and an ipsilateral or bilateral CST wiring, which led to increased sensory deficits (*p* < 0.05). These novel insights contribute to a better understanding of the underlying pathophysiology of UL function and may be useful to delineate individualized treatment strategies.

## 1. Introduction

Upper limb (UL) function is commonly impaired in individuals with unilateral cerebral palsy (uCP), negatively impacting on daily life activities [1]. The large variability in the clinical presentation of UL function, but also in treatment response, has resulted in increasing interest in understanding the underlying neural mechanisms that determine UL function and its contribution to further optimize therapy planning for the individual with uCP. A number of neurological factors have been put forward as potential predictors of UL function, i.e., the structural brain lesion characteristics (i.e., lesion timing, location, and extent), and the type of corticospinal tract (CST) wiring [2–6].

The timing of the lesion during gestation is closely related to the type of the damaged tissue and can be classified into three categories: malformations (1^st^ and 2^nd^ trimesters of pregnancy), periventricular lesion (PV, early 3^rd^ trimester), and corticosubcortical lesions (CSC, late 3^rd^ trimester and around birth) [7]. Previous studies investigating the impact of lesion timing on UL function have shown that individuals with a later lesion (i.e., CSC lesions) present with poorer UL motor and sensory functions [2, 3, 5]. Besides lesion timing, lesion location and extent have shown to play an important role in determining UL function, whereby damage to the posterior limb of the internal capsule (PLIC) and the basal ganglia, and a larger lesion extent is related to worse UL motor and sensory functions [2, 3]. However, there is still large variability in UL function that remains unexplained based on these factors.

The unilateral brain damage in individuals with uCP can also result in a partial or complete reorganization of the CST toward the nonlesioned hemisphere [8]. This reorganization of the CST wiring is unique in uCP and refers to the efferent motor input to the affected hand. Researchers have identified three types of CST wiring, i.e., contralateral (CST_contra_, the affected hand receives input from the crossed CST, originating in the lesioned hemisphere), ipsilateral (CST_ipsi_, the affected hand receives input from the uncrossed CST, originating in the nonlesioned hemisphere), and bilateral (CST_bilat_, the affected hand receives input from both the crossed and uncrossed CSTs, originating in the lesioned and nonlesioned hemispheres, respectively) [8, 9]. It has been suggested that the type of CST wiring is the main factor influencing UL function, whereby individuals with CST_contra_ present with more preserved UL function compared to the other groups [6, 10–13]. Nevertheless, assessing the underlying CST wiring with Transcranial Magnetic Stimulation (TMS) in young children might become challenging. Therefore, the identification of either behavioural or brain lesion features that relate to the underlying CST wiring could be useful to define tailor-made interventions in a clinical setting.

Whilst the role of lesion timing, location, and extent has been well investigated [2, 3, 14], only a few studies examined the impact of the CST wiring on UL function and they often have several limitations (i.e., small sample sizes, ordinal scoring of impairments, and limited to motor deficits) [5, 10, 15]. Moreover, studies thus far focused on each factor independently, whereas only one study described the impact of the CST wiring and lesion timing on UL function in uCP [10], and only one study reports the impact of CST wiring and lesion extent in children with PV lesions [4]. Although the authors suggested the relevance of both lesion timing and type of CST wiring in predicting UL function, the small sample size, the lack of a standardized evaluation of motor function, and the merely descriptive nature of the study hampered the possibility of drawing strong conclusions.Furthermore, it has been shown that an intact sensory function is essential to develop an adequate motor function in other neurological disorders (such as adult stroke) [16, 17]. Also in individuals with uCP, sensory and motor functions are highly related [1], although the impact of the CST wiring on this relationship remains unknown.

In this study, we investigated the impact of CST wiring and structural brain lesion characteristics on UL motor and sensory functions in a large group of individuals with uCP, using a systematic and comprehensive evaluation. Our first hypothesis is that the type of the CST wiring pattern in unilateral CP can be predicted based on a linear combination of measures of lesion timing, location, and extent. Second, we hypothesize that the combination of these predictors together with the CST wiring has a stronger predicting value for UL motor and sensory functions than any of these factors alone. Last, we speculate that the relation between motor and sensory functions is disrupted by the type of CST wiring.

## 2. Materials and Methods

### 2.1. Participants

Children and adolescents with uCP aged between 5 and 21 years old were recruited via the CP reference center of the University Hospitals Leuven between 2014 and 2017. They were excluded if they (1) received UL botulinum toxin injections six months prior to the assessment, (2) had UL surgery two years prior to the assessment, and/or (3) had other neurological or genetic disorders. All individuals assented to participate; all parents signed the informed consent (participants younger than 18 years old), and participants older than 12 years also signed the informed consent, in accordance with the Declaration of Helsinki. This study was approved by the Medical Ethical Committee of the University Hospital Leuven (S55555 and S56513).

Participants with contraindications for the MRI (e.g., metal implants) or the Transcranial Magnetic Stimulation (TMS; ventricular-peritoneal (VP) shunt, seizure two years prior to the study) did not undergo the respective assessment. All TMS measurements were conducted by two experienced physiotherapists (CSM and EJ), and UL function was evaluated by four experienced physiotherapists (LM, CSM, JH, and EJ) at the Clinical Motion Analysis Laboratory of the University Hospitals Leuven (campus Pellenberg, Belgium).

### 2.2. Upper Limb Evaluation

#### 2.2.1. Motor Function

Grip strength, unimanual capacity, and bimanual performance composed the motor evaluation. Maximum *grip strength* was assessed using the Jamar® hydraulic hand dynamometer (Sammons Preston, Rolyan, Bolingbrook, IL, USA). The less-affected hand was measured first, and the mean of three maximum contractions was calculated per hand. The ratio between hands was used for further analyses to cancel out the effect of age (grip strength ratio = grip strength less − affected hand/grip strength affected hand, whereby a lower score (closer to 1) indicates a grip strength in the affected hand similar to that of the less-affected hand). *Unimanual capacity* was assessed with the Jebsen-Taylor hand function test (JTHFT). The JTHFT reliably measures movement speed during six unimanual tasks [18, 19]. Similar to other studies, we used a modified version for children and adolescents with uCP in which the writing task was removed and the time to carry out each task was reduced from 3 to 2 minutes to avoid frustration [19, 20]. The time to perform every task was summed up, and the ratio between hands was used for further analyses to cancel out the effect of age (JTHFT ratio = JTHFT affected hand/JTHFT less-affected hand, whereby a lower score (closer to 1) indicates movement speed in the affected hand similar to that of the less-affected hand). *Bimanual performance* was evaluated with the Assisting Hand Assessment (AHA), which assesses how effectively the affected hand is used in bimanual activities [21–23]. The spontaneous use is evaluated during a semistructured play session with standardized toys requiring bimanual handling. Given the age range of the participants of this study, the School Kids AHA and the Ad-AHA were administered [22, 24]. The AHA was scored by certified raters (LM and CSM), using the 5.0 version which includes 20 items that are scored from 0 (“does not do”) to 4 (“effective use”), resulting in a final score between 0 and 100 AHA units.

#### 2.2.2. Sensory Function

Sensory assessments comprised measures of exteroception (tactile sense), proprioception (movement sense), two-point discrimination (2PD, Aesthesiometer®), and stereognosis (tactile object identification), which have been shown to be reliable in this population [25]. Tactile and movement senses were classified as normal (score 2), impaired (score 1), or absent (score 0). 2PD was classified according to the width between the two points that the participants could discriminate: normal (0–4 mm, score 2) or impaired (>4 mm, score 1) [26]. Tactile object identification was used as the number of objects that the children could recognize (0–6). In addition, a kit of 20 nylon monofilaments (0.04 g–300 g) (Jamar Monofilaments, Sammons Preston, Rolyan, Bolingbrook, IL, USA) was used to reliably determine threshold values for touch sensation [27, 28]. Touch sensation was categorized as normal (0.008–0.07 g), diminished light touch (0.16–0.4 g), diminished protective sensation (0.6–2 g), loss of protective sensation (4.19–180 g), and untestable (300 g), according to the manual (Jamar Monofilaments, Sammons Preston, Rolyan, Bolingbrook, IL, USA).

### 2.3. Structural MRI

Structural images were acquired using three-dimensional fluid-attenuated inversion recovery (3D FLAIR) (321 slices, slice thickness = 1.2 mm, slice gap = 0.6 mm, repetition time = 4800 ms, echo time = 353 ms, field of view (FOV) = 250 × 250 mm^2^, 1.1 × 1.1 × 0.56 mm^3^ voxel size, acquisition time = 5 minutes). In addition, magnetization prepared rapid gradient echo (MPRAGE) was acquired (182 slices, slice thickness = 1.2 mm, slice gap = 0 mm, TR = 9.7 ms, TE = 4.6 ms, FOV = 250 × 250 mm^2^, voxel size = 0.98 × 0.98 × 1.2, acquisition time = 6 minutes). The structural MRI was used to provide a detailed description of the lesion location and extent and to classify the timing of the lesion, which was conducted by a paediatric neurologist (EO).

Timing of the brain lesion was classified according to the predominant pattern of damage as described by Krägeloh-Mann and Horber [7]: malformations (1^st^ and 2^nd^ trimesters of pregnancy), periventricular lesion (PV, early 3^rd^ trimester), corticosubcortical lesions (CSC, late 3^rd^ trimester and term), or acquired brain lesions (between 28 days and two years postnatally).

Lesion location and extent were determined using a semiquantitative scale recently developed by Fiori et al. [29]. The scale consists of a graphical template with six axial slices of the brain and an extra template for the basal ganglia (lenticular and caudate), thalamus, posterior limb of the internal capsule (PLIC), brainstem, corpus callosum, and cerebellum. Firstly, the slices corresponding to the template slices are to be found and the lesion is drawn onto the template. Next, the damage to the periventricular, middle, and corticosubcortical layers of each lobe is scored for both hemispheres separately. The sum of the damage to each lobe results in the lobar score, ranging from 0 to 3 for each lobe. Damage to the basal ganglia (lenticular and caudate), thalamus, PLIC, and brainstem directly is binarily scored from the MRI (affected or nonaffected). Damage to the corpus callosum is scored from 0 to 3, based on the involvement of the anterior, middle, and posterior thirds of the corpus callosum on a sagittal view. Last, the involvement of the cerebellum is based on damage to the vermis (0–1) and each of the hemispheres (0–2), resulting in a total score ranging from 0 to 3. A total ipsilesional score is calculated based on the damage to the lobes (0–3 for each lobe, i.e., total of 0–12) and damage to the subcortical structures (0–5; ranging from 0 to 17). More detailed information about the scale and its scoring procedure can be found in the respective study [29]. This semiquantitative scale has been shown valid and reliable in children with uCP [29, 30].

In the present study, lesion location was indicated by the damage to the frontal and parietal lobes (0–4), damage to the basal ganglia and thalamus (0–3), and damage to the PLIC (0–1). These locations were chosen based on their relation to the sensorimotor system [31]. Lesion extent was indicated by the total ipsilesional score (0–17).

### 2.4. Transcranial Magnetic Stimulation

Single-pulse TMS was conducted to assess CST wiring. TMS was applied using a Magstim 200 stimulator (Magstim Ltd., Whitland, Wales, UK) equipped with a focal 70 mm figure-eight coil and a Bagnoli electromyography (EMG) system with two single differential surface electrodes (Delsys Inc., Natick, MA, USA). A Micro1401-3 acquisition unit and Spike software version 4.11 (Cambridge Electronic Design Limited, Cambridge, UK) were used to synchronize the TMS stimuli and the EMG data acquisition. Motor evoked potentials (MEPs) were bilaterally recorded from the muscles opponens pollicis brevis. During the TMS assessment, participants wore a cap that allows creating a grip with a coordinate system to identify the optimal point to stimulate (hotspot) in a standardized and systematic way. The hotspot and the resting motor threshold (RMT, defined as the minimum intensity required to obtain 5/10 MEP of at least 50 *μ*V in the corresponding muscle) were identified by starting the stimulation intensity at 30% with an incremental increase of 5% [4]. For each hemisphere, stimulation started from the assumed “motor hotspot,” which is located 5 cm lateral and 1 cm anterior from the scalp middle point (Cz), at 30%. After approximately 2–3 pulses, the stimulation intensity was increased 5% for another 2–3 pulses, until MEPs were found. If no MEP can be elicited after increasing up to 60 to 80%, the coil would be moved to a different location on the scalp grid and the procedure would be repeated until an MEP was elicited. Stimulation up to 100% of the maximum stimulator output was continued until an MEP was elicited. The nonlesioned hemisphere was always stimulated first and allowed to identify contralateral CST projections to the less-affected hand. Stimulation in the nonlesioned hemisphere was continued up to 100% of the maximum stimulator output to search for possible ipsilateral CST projections to the affected hand. Next, the lesioned hemisphere was stimulated to identify possible contralateral CST projections to the affected hand. If only contralateral MEPs from each hemisphere were found, the child was categorized as having a CST_contra_ wiring. If MEPs in the affected hand were evoked from both hemispheres, the child was categorized as having a CST_bilat_ wiring. Lastly, if MEPs in the impaired hand were only evoked when stimulating the nonaffected hemisphere, the child was categorized as having a CST_ipsi_ wiring. TMS measures have been shown to be reliable in adults [32, 33] and in children [34]. In this study, the TMS assessment was used for diagnostic purposes. In cases when high intensities were not tolerated, the stimulation intensity was increased up to at least 80% of the maximum stimulator output and children were asked to hold a pen to ensure precontraction of the evaluated muscle and thereby facilitate the CST and MEP detection. This allowed us to rule out the possibility of miscategorizing the child regarding their CST wiring pattern.

### 2.5. Statistical Analyses

First, descriptive statistics were used to document the distribution of brain lesion characteristics according to the CST wiring. Next, we investigated the differences in occurrence of lesion timing, location, and extent between the CST wiring groups by using analysis of contingency tables (chi-square and Fisher's exact tests), Kruskal-Wallis test (ordinal data), and ANOVA (lesion extent). Lastly, we used discriminant analysis to explore whether the type of CST wiring would differ depending on the linear combination of lesion timing, location, and extent, in a multivariate way. Cross-validation procedure was included to investigate the accuracy of the model in reclassifying the participants in the original CST wiring groups. Variables related to lesion timing, lesion location (damage to the frontal lobe, parietal lobe, PLIC, basal ganglia, and thalamus), and extent (ipsilesional extent of the lesion) were included in the model, which was fitted using the stepwise selection method.

To investigate the impact of the type of CST wiring and brain lesion characteristics on UL function, we first used linear simple regression and then multiple regression analysis to investigate the combined impact of these factors on UL motor and sensory functions. For the continuous variables related to motor function, normality was first verified by inspecting the histograms and with the Shapiro-Wilk test, showing a normal distribution only for the AHA. For the JTHFT ratio and the grip strength ratio, a logarithmic transformation was applied (*y*′ = log10 (*y*)). To investigate the impact of the type of CST wiring and brain lesion characteristics on UL motor function, we computed a multiple regression analysis. Similarly, for UL sensory function, we conducted a simple ordinal logistic regression for stereognosis and thresholds for touch sensation and a simple logistic regression for 2PD to investigate the impact of each individual neurological factor on the sensory function. Next, we performed multiple regression analyses (ordinal and logistic) to investigate the combined impact of the neurological predictors on the sensory deficits. The predictors included in the multiple regression model were the type of CST wiring, lesion timing, location (damage to the frontal lobe, parietal lobe, PLIC, basal ganglia, and thalamus), and ipsilesional extent of the lesion. To predict both motor and sensory functions, interaction terms were built between the CST wiring and (i) lesion timing and (ii) lesion extent and included in the model. The multiple regression models were fitted with the backward elimination method until a set of variables significantly contributing to the model was identified.

Lastly, to investigate the relation between sensory and motor functions for the whole group and within CST wiring groups, Spearman rank correlation coefficients were used between each of the motor function variables and deficits in stereognosis. Correlation coefficients were considered as little or no correlation (<0.30), low (0.30–0.50), moderate (0.50–0.70), high (0.70–0.90), and very high correlation (>0.90) [35].

In addition, effects sizes were calculated for the comparisons and interpreted according to Cohen, depending on the computed test: *η* ^2^ (partial eta squared) for the prediction models (small 0.01, medium 0.06, and large 0.14) [36, 37]. Statistical significance was set at *α* < 0.05 for main tests with Bonferroni correction for post hoc tests. All statistical analyses were computed with SPSS Statistics for Windows version 24.0 (IBM Corp., Armonk, NY).

## 3. Results

### 3.1. Participants

Seventy-five children and adolescents with uCP participated in this study (mean age (SD): 11 y and 1 m (3 y and 6 m); 33 girls; 39 left uCP). According to the Manual Ability Classification System (MACS), 25 individuals were classified as MACS I, 25 as MACS II, and 25 as MACS III. Sixteen participants did not have CST wiring data (*n* = 1 panic attack, *n* = 2 hemispherectomy, *n* = 3 VP shunt, *n* = 2 epilepsy, *n* = 1 tumor, *n* = 4 refusals to participate, and *n* = 3 inconclusive TMS results), resulting in a total of 59 participants. The TMS assessment identified 20 individuals with CST_contra_, 18 with CST_bilat_, and 21 with CST_ipsi_. For the analyses in this study, participants with malformations (*n* = 1), acquired lesions (*n* = 4), or no visible lesions (*n* = 2) were excluded due to the very small sample size of these subgroups, resulting in a total group of 52 participants (mean age (SD): 11 y and 4 m (3 y and 10 m); 22 girls; 28 left uCP) with available CST wiring (*n* = 17 contralateral, *n* = 19 ipsilateral, and *n* = 16 bilateral) and data related to the timing, location, and extent of the lesion. A summary of the lesion locations and extent according to the lesion timing is provided in Supplementary Materials (Table 1). Thirty-four individuals had a PV lesion, and 18 had a CSC lesion. Clinical motor and sensory data was missing in one participant (boy, 19 y and 7 m, PV lesion, and CST_contra_ wiring), and sensory data was evaluated in a subsample of participants (see Section 3.3.2 for more details).

### 3.2. CST Wiring and Brain Lesion Characteristics

Table 1 displays the distribution of lesion timing, location, and extent variables according to the three CST wiring groups. Except for the damage to the parietal lobe, all variables were significantly different between the CST wiring groups (*p* < 0.05) (Table 1).

In the discriminant analysis, we found that the combined value of the damage to the PLIC and the damage to the frontal lobe could significantly discriminate between the type of CST wiring (Wilks' *λ* = 0.611, chi-square test = 23.88, df = 4, canonical correlation = 0.602, *p* < 0.001). The two functions extracted accounted for nearly 57% of the variance in the type of CST wiring. The standardized discriminant function coefficients of the two extracted functions indicated the contribution of each retained independent variable (damage to the PLIC and damage to the frontal lobe) to each function, showing how strongly the discriminant variables affect the score. These coefficients can be then used for the classification of a single individual (function 1 = 0.81 ∗ damage to the PLIC + 0.50 ∗ damage to the frontal lobe; function 2 = −0.60 ∗ damage to the PLIC + 0.88 ∗ damage to the frontal lobe).

**Figure 1:**
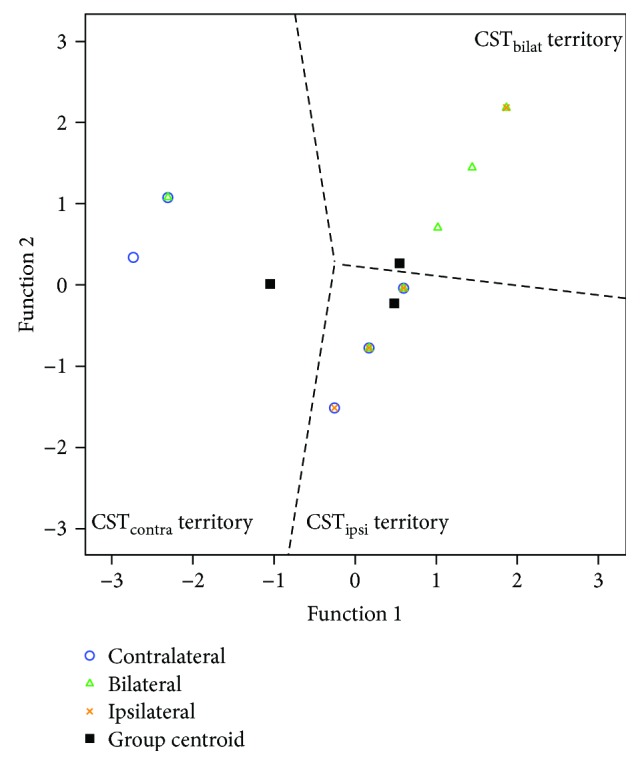
Territorial map showing the relative location of the boundaries of each CST wiring category and the location of each of the participants. The group centroids are indicated with a black-filled square (CST_contra_ (−1.05, 0.01), CST_ipsi_ (0.48, −0.23), and CST_bilat_ (0.54, 0.26)).

Cross-validated reclassification of cases based on the new canonical variables was successful in 57.7% of the cases: 89.5% were correctly classified in the CST_ipsi_ group, 47.1% in the CST_contra_ group, and only 31.3% in the CST_bilat_ group (Figure 1).

### 3.3. CST Wiring, Brain Lesion Characteristics, and UL Function

#### 3.3.1. Motor Function

Descriptive statistics of the motor function according to the type of CST wiring, lesion timing, location, and extent are presented in Supplementary Materials (Table 2). The simple linear regression analyses to predict motor function based on a single neurological factor showed that every factor had an influence on motor function (grip strength, *p* < 0.04; JTHFT, *p* < 0.004; AHA, *p* < 0.01; see Supplementary Materials Table 2 for detailed information).

**Figure 2:**
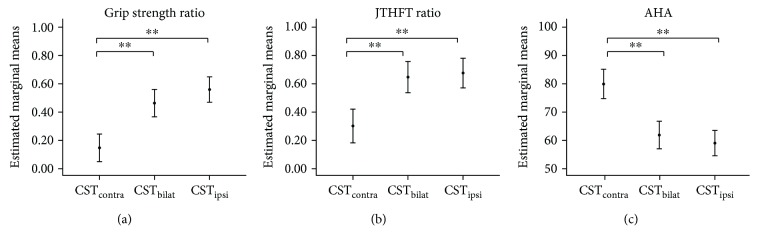
Upper limb motor function differs in individuals with CST_contra_ wiring compared to those with CST_bilat_ or CST_ipsi_ wiring. Estimated marginal means and 95% CI per CST wiring type and lesion timing group for (a) grip strength (log ratio, i.e., closer to zero indicates preserved grip strength), (b) JTHFT (log ratio, i.e., closer to zero indicates preserved manual dexterity, measured by speed), and (c) AHA. AHA: Assisting Hand Assessment; JTHFT: Jebsen-Taylor hand function test; CST: corticospinal tract. ^∗^ *p* < 0.01; ^∗∗^ *p* < 0.001. Estimated marginal means are adjusted according to the significant covariates (see Table 2 for details).

When all the neurological factors were included in the same model in a multiple regression analysis, the backward elimination method identified the variables that were significantly contributing to the model.  Table 2 documents the estimated marginal means, which represent the mean response in each CST wiring group adjusted by the covariates that significantly contribute to the model. The multiple regression model to predict grip strength deficits only retained the type of CST wiring, explaining 46% of the variance (*F*(2, 51) = 20.90; *p* < 0.001; *η* ^2^ = 0.47). For the JTHFT, 54% of the variance was explained by the type of CST wiring (*F*(2, 51) = 12.20; *p* < 0.0001; *η* ^2^ = 0.34, *R* ^2^ = 46%) and the total extent of the lesion (*F*(1, 51) = 8.05; *p* = 0.007; *η* ^2^ = 0.15, Δ*R* ^2^ = 8%). For bimanual performance (AHA), the regression model explained 61% of the variance, with the type of CST wiring (*F*(2, 51) = 19.03; *p* < 0.0001; *η* ^2^ = 0.45, Δ*R* ^2^ = 52%), the total extent of the lesion (*F*(1, 51) = 10.65; *p* < 0.001; *η* ^2^ = 0.19, Δ*R* ^2^ = 5%), and the damage to the basal ganglia and thalamus (*F*(1, 51) = 4.90; *p* = 0.03; *η* ^2^ = 0.10, Δ*R* ^2^ = 4%) significantly contributing to the model (Figure 2). No interaction effects were identified for any of the motor outcome variables.

#### 3.3.2. Sensory Function

Descriptive information of sensory function according to each neurological factor is summarized in Table 3 of Supplementary Materials. Sensory function data (tactile sense, movement sense, stereognosis, and 2PD) and thresholds for touch sensation, as assessed with the monofilaments, were available in 46 and 35 individuals, respectively. Due to the lack of variation in the tactile sense and movement sense modalities, the predictive model was only applied to the stereognosis, 2PD, and the thresholds for touch sensation.

The simple linear analyses to predict sensory function based on a single neurological predictor indicated that every predictor impacted on stereognosis (*p* < 0.032). In contrast, 2PD was influenced by all neurological predictors (*p* < 0.04) except the damage to the PLIC (*p* < 0.17) and touch sensation could be significantly predicted by all factors (*p* < 0.01) except damage to the PLIC (*p* = 0.99) and type of CST wiring (*p* = 0.42).

When all the neurological factors were included in the same model in a multiple regression analysis, the backward elimination method identified predictors that were significantly contributing to the model. For stereognosis, the retained main effects were the CST wiring (Wald chi-square test (2) = 9.09, *p* = 0.011), lesion timing (Wald chi-square test (1) = 4.34, *p* = 0.04), and ipsilesional extent of the lesion (Wald chi-square test (1) = 7.15, *p* = 0.008) (Table 3(a)). These results show that the odds of having better stereognosis function were 5.56 times higher in the group with PV lesions than in the CSC group (*p* = 0.04). Similarly, individuals with a CST_contra_ wiring show 10.23 and 9.7 times higher probability of having better scores in the stereognosis test compared to those with a CST_ipsi_ or CST_bilat_ wiring, respectively (*p* = 0.02), whilst there was no difference between the last two (*p* = 0.34). Lastly, the odds of having higher stereognosis scores decrease by 0.74 for every unit change in the ipsilesional extent of the lesion (*p* = 0.01). No interactions were found between the CST wiring and the brain lesion characteristics to predict deficits in stereognosis (*p* > 0.05).

The logistic multiple regression to predict 2PD showed lesion timing (Wald chi-square test (1) = 10.62, *p* = 0.001) and ipsilesional extent of the lesion (Wald chi-square test (1) = 3.75, *p* = 0.05) to be significant contributors (*p* > 0.05) (Table 3(b)). The odds of having an impaired 2PD are 31 times higher in the group with CSC lesions than in the PVL group (*p* = 0.001). Secondly, the odds of having impaired 2PD increase by 1.34 for every unit change in the ipsilesional extent of the lesion (*p* = 0.05). No interactions were found between the CST wiring and the brain lesion characteristics to predict deficits in 2PD (*p* > 0.05).

The ordinal logistic multiple regression for touch sensation, as measured by the monofilaments, indicated that only the lesion extent significantly contributed to the deficits in touch sensation (Wald chi-square test (1) = 10.75, *p* = 0.001) (Table 3(c)). The odds of having better touch sensation decrease by 0.66 for every unit change in the ipsilesional extent of the lesion. No interactions were found between the CST wiring and the brain lesion characteristics to predict deficits in touch sensation (*p* > 0.05).

#### 3.3.3. Impact of CST Wiring on the Relation between Motor and Sensory Functions

The correlation analyses between the motor and sensory functions for the whole group indicated a moderate association between the stereognosis score and grip strength ratio (*r*_*s*_ = −0.60, *p* < 0.001), JTHFT ratio (*r*_*s*_ = −0.60, *p* < 0.001), and AHA (*r*_*s*_ = 0.61, *p* < 0.001).

**Figure 3:**
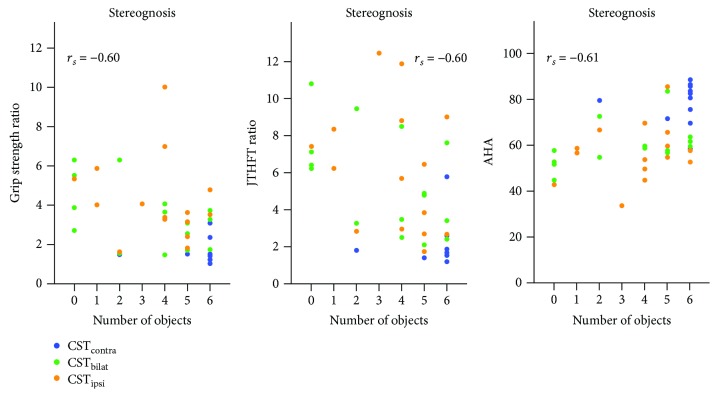
The relation between motor and sensory functions seems to vary depending on the CST wiring. Individuals with a CST_contra_ and CST_ipsi_ wiring showed no low correlations, whereas those with CST_bilat_ showed moderate correlations. Each dot represents an individual child, with CST_contra_ (blue), CST_bilat_ (green), and CST_ipsi_ (orange). Correlations between stereognosis with grip strength ratio (ratio, i.e., closer to one indicates preserved grip strength), JTHFT ratio (ratio, i.e., closer to one indicates preserved grip strength), and AHA. Correlation coefficients correspond to the analysis for the whole group.

After group division according to CST wiring, there was no low correlation between motor function and stereognosis in the CST_contra_ and CST_ipsi_ groups (*r*_*s*_ (range) = −0.31–0.36, *p* > 0.05). Interestingly, in the CST_bilat_ group, moderate correlations were found with the JTHFT ratio (*r*_*s*_ = −0.48, *p* = 0.07) and the AHA (*r*_*s*_ = 0.65, *p* < 0.01), despite a low correlation with grip strength ratio (*r*_*s*_ = −0.31, *p* = 0.2). An illustration of the individual data points regarding these results can be found in Figure 3.

## 4. Discussion

In this study, we explored the predictive value of brain lesion characteristics on the type of CST wiring as well as the impact of these factors on UL motor and sensory functions. A comprehensive and standardized evaluation of both motor (grip strength, unimanual capacity, and bimanual performance) and sensory functions was used to predict UL function in a large cohort of individuals with uCP.

Our first research question examined the discriminant ability of lesion timing, location, and extent to predict the type of CST wiring. A simple linear analysis demonstrated that lesion timing, location, and extent were significantly different between the CST wiring groups. Our results showed that a CST_contra_ was only seen in 2 out of 18 children with a CSC lesion, compared to 15 out of 34 children with a PV lesion. Current results suggest that damage to cortical and/or subcortical structures (i.e., CSC lesion) reduces the potential of the CST to develop according to its typical contralateral trajectory. We hypothesize that this is likely driven by the reduced neural activity in the motor cortical areas after a CSC lesion, which are crucial for the development of the CST during the postnatal period [38]. However, a contralateral development of the CST is still possible in CSC lesions, and it may occur differently depending on lesion location and extent.

Once all predictors were simultaneously entered in a multiple linear analysis, we found that the combination of the damage to the PLIC and the frontal lobe significantly discriminated between the CST wiring groups. Half of the children in the CST_contra_ group showed damage to the PLIC, in contrast to the 94% and 100% in the CST_bilat_ and CST_ipsi_ groups who showed damage to this white matter bundle. Furthermore, the frontal lobe was also more damaged in the CST_bilat_ and CST_ipsi_ groups, compared to the CST_contra_ group. Although it is not unexpected that the PLIC and the frontal lobe are the two significant predictors in the model, due to their undoubtable relation with the motor cortex and the performance of actions, this is the first time that this interaction with the type of CST wiring is shown. Contrary to the importance of the location, Staudt et al. [4] postulated that the type of CST wiring depended on the lesion extent. However, as they only included children with a PV lesion, their results cannot be extended to all the uCP populations. Further efforts should be made to underpin whether structural damage of the brain lesion may serve as a biomarker of the underlying CST wiring.

Next to the predictive model, we also investigated how accurate the two functions derived from the discriminant analysis would be to reclassify the individuals in their original categories. Despite the significant contribution of the PLIC and the frontal lobe to the discriminant model, the classification accuracy only reached 57%, suggesting that timing, location, and extent of the lesion (as included in the model) do not provide sufficient accurate information to predict the underlying type of CST wiring. Notwithstanding the validity and reliability of the semiquantitative scale that was used to investigate lesion location and extent, we acknowledge that the semiquantitative character of the scale may have underestimated the predictive value of the structural brain damage. Therefore, these results should be replicated in the future with volumetric measures of the different brain structures. For example, the projections to the PLIC have been shown to be topographically organized with reduced microstructural integrity in children with uCP [39] by using diffusion measures. Investigating the volumetric damage to the frontal lobe and the microstructural integrity of the PLIC may provide with further insights in determining the type of CST wiring in uCP.

For our second research question, we investigated the impact of CST wiring and brain lesion characteristics (timing, location, and extent) on motor and sensory functions. Regarding *motor outcome*, simple linear regression analyses indicated that the CST wiring and all brain lesion characteristics had an influence on the grip strength, manual dexterity, and bimanual performance, which confirmed what previous studies have shown [5, 6, 10]. However, in the multiple linear regression analysis, we found that the underlying CST wiring plays a major, but not unique, role in determining UL motor function, as lesion location and extent also significantly contributed to increasing the explained variance for the JTHFT and AHA. Specifically, the type of CST wiring explained 46% and 52% of the JTHFT and AHA variances, respectively, which was increased up to 54% and 61% by including lesion extent and damage to the basal ganglia and thalamus into the model. In general, our results show that a CST_ipsi_ or CST_bilat_ leads to poorer UL motor function compared to CST_contra_ for all motor outcomes, even when controlling for the significant contribution of lesion extent and location. The importance of the underlying CST wiring is an expected result, as the CST is the main motor drive and its damage causes vast disturbances on voluntary motor control, drastically reducing motor capabilities [38]. Whilst lesion timing, location, and extent have been put forward as a predictor of UL function [2, 3] and were also confirmed in our linear regression analysis, the huge variability in motor function reported by previous studies seems to be mainly explained by the underlying CST wiring. Staudt et al. [10] were the first to report on the relation between CST reorganization potential at different gestational ages and UL motor function. These authors also found that, along with the CST wiring, UL motor function further worsened in later lesions (CSC lesions) [10]. Linear regression analysis also showed that later lesions led to poor motor outcome, but multiple regression analysis revealed that lesion location and extent were key factors, next to the type of CST wiring. Although later lesions seem to be associated to a larger extent [3], it seems that the lesion extent itself plays a more important role in motor outcome, i.e., children with a PV lesion with large extent will also present with poorer hand function. Interestingly, the damage to the basal ganglia and thalamus explained an extra 4% of the variability in the AHA. In accordance with our results, previous studies have reported the negative impact of these subcortical structures on UL motor outcome [2, 5].

It is important to note that we still found large variability in the three motor outcome measures within both the CST_ipsi_ and CST_bilat_ groups, whereas the variability in the CST_contra_ group was rather small (Figure 2, see also Table 2 Supplementary Materials for observed means). In other words, some individuals with a CST_ipsi_ and CST_bilat_ wiring had good motor function, similar to those with a CST_contra_ wiring. This variability could not be completely explained by the location and extent of the lesion, and other factors may play a role. In the CST_ipsi_ group, this large variability may be explained by the amount of overlap of the hotspot within the nonlesioned hemisphere to evoke MEPs in the affected and less-affected hands. Vandermeeren et al. [40] showed that dexterity indeed varies in individuals with ipsilateral wiring depending on the location of the hotspot of the CST innervating the affected hand and less-affected hand; overlapping hotspots resulted in poorer dexterity, whereas distinct nonoverlapping hotspots resulted in a preserved dexterity. Conversely, in the CST_bilat_ group, the large variability may be explained by a predominant contralateral or ipsilateral projection that controls the affected hand, as Jaspers et al. [9] proposed in their theoretical framework. Altogether, this seems to point toward a distinct underlying pathophysiology of the UL motor impairments in these two CST groups (CST_ipsi_ or CST_bilat_), suggesting that individuals with either a CST_bilat_ or CST_ipsi_ pattern should be treated as two separate groups for future research. To further unravel the underlying mechanisms of the pathophysiology of motor control and motor capabilities in uCP, additional functional measures should be included such as excitatory and inhibitory intracortical circuits based on TMS (e.g., cortical silent period or paired-pulse paradigms) [15, 41] or functional connectivity of the sensorimotor network based on resting-state functional MRI [42, 43].

We also investigated the impact of the CST wiring and brain lesion characteristics on *sensory function*, based on the fact that CST projections also extend from the primary sensory cortex and mediate several sensory functions at the level of the spinal cord (control of nociceptive, somatosensory, and somatic motor functions) [44, 45]. Although our simple linear regression analyses suggested that all neurological factors individually played a role in determining sensory function, the multiple prediction model showed that a larger lesion extent, a later lesion (i.e., CSC lesion), and a CST_ipsi_ or CST_bilat_ led to higher chances of developing sensory deficits. Our results are in agreement with a recent study by Gupta et al. [6], who showed that more than 80% of the children with larger extent and later lesions (CSC) had disrupted somatosensory anatomy and physiology (lack of ascending sensory tracts and lack of somatosensory evoked potentials), consequently leading to a loss of sensory function [6]. If the sensory tracts are present, there is evidence suggesting that their main compensatory mechanism is an intrahemispheric reorganization, i.e., the sensory system reaches the original cortical destination on the postcentral gyrus, regardless of lesion timing (PV or CSC lesion) or CST wiring [11, 46, 47]. Current study results suggest that lesion extent best predicts the sensory deficits in individuals with uCP, although lesion timing and CST wiring also play an important role. Future research focusing on the pathophysiology of the sensory system based on noninvasive neurophysiological techniques (e.g., short-latency afferent inhibition [48] or sensory evoked potentials [11]), as well as functional connectivity measures, may contribute to increase our understanding of the underlying sensory pathways in uCP.

Lastly, we investigated whether the relationship between motor and sensory functions was disrupted by the type of CST wiring. We first confirmed previous study results indicating a significant relation between the motor and sensory outcomes in the total group [1, 25]. However, this association was disrupted by the type of CST wiring, whereby no little association was shown in the CST_ipsi_ and CST_contra_ groups, but a moderate association was found for the CST_bilat_ group. In the CST_contra_ group, the lack of a significant (or high) correlation seems to be due to the fact that these participants show both adequate motor and sensory functions, with little variation in the sensory scale, due to its ordinal nature. This scale used to evaluate sensory function may not be sensitive enough to detect subtle sensory deficits, leading to a possible ceiling effect in the CST_contra_ group. By measuring with more quantitative techniques and devices, e.g., KINARM End-Point Lab (BKIN Technologies) [49], we may be able to discern the potential sensory problems that these individuals may present with. Secondly, the sensorimotor dissociation found in the CST_ipsi_ group may be explained at two different levels of the central nervous system. At the level of the spinal cord, the descending CST fibres entering the dorsal horn play an important role in presynaptic inhibition of primary sensory afferent fibres [45, 50], ensuring smooth execution of a movement. A CST_ipsi_ wiring may have consequences in the presynaptic inhibition at the level of the spinal cord and could, consequently, affect the relation between motor and sensory functions. On the other hand, at the level of the brain, the intrahemispheric communication between M1 and S1 has been shown to be very relevant for adequate processing of sensorimotor information [51–53]. As such, the lack of intrahemispheric corticocortical connections may affect the processing of sensory information, having a negative impact on the motor command. On the contrary, the CST_bilat_ group seems to preserve the relation between motor and sensory functions, as shown by the stereognosis modality. This may be potentially explained by the predominant behaviour that those with a CST_bilat_ wiring hypothetically show [9]. A relation between adequate sensory and adequate motor functions, as seen in the CST_contra_ group, may indicate a more “contralateral” behaviour, whilst a disparate relation may be indicative of rather an “ipsilateral” behaviour. However, this needs further confirmation with neurophysiological tools. Although current data do not allow drawing strong conclusions regarding sensorimotor integration, our results highlight the importance of investigating these aspects in the future to better understand the mechanisms of sensorimotor information processing in uCP. By using more advanced techniques to unravel the coupling between the sensory and motor systems, we will be able to determine the impact of such dissociation on motor control and motor performance. For instance, short-latency afferent inhibition has been put forward as a valuable indicator of the process of bilateral sensorimotor integration [48] and may potentially aid in measuring the reorganization of sensorimotor pathways in uCP.

There might be some important clinical implications based on the results of this study. A better understanding of the underlying mechanisms of motor and sensory impairments will surely contribute to developing new treatment approaches, specifically targeting the individual pathophysiological deficits. First, the type of CST wiring has been investigated as a potential biomarker of treatment response. Although motor improvement does not seem to be CST-type dependent after bimanual training [12, 54], there are conflicting results regarding unimanual training [55–57]. Furthermore, our results highlight the importance of considering the sensory system together with the available motor execution paradigms during UL training. Preliminary results of recent studies have shown the effectiveness of bimanual and sensory training on both motor and sensory functions in uCP [58, 59]. To further support interventions targeting sensory deficits, there is evidence in healthy adults suggesting that sensory input can modulate the excitability in both motor cortices simultaneously, as well as the communication between hemispheres [60]. In this line, it seems relevant to combine bimanual and sensory training to enhance the excitability of both motor cortices, which may increase intra- and interhemispheric connections between the sensory and motor systems, potentially resulting in long-lasting neuroplastic changes.

Next to the training approaches, it is also important to identify clinically feasible measures to infer the CST wiring and the sensory system. As these assessments are not always pleasant in young children nor practical in a clinical setting, there is a necessity to find tools that are more applicable to daily practice than neurophysiological techniques. To probe the motor system, mirror movements have been put forward as a valid clinical assessment tool that may reflect the underlying individual CST wiring [9, 61]. On the other hand, it seems very challenging to develop an accessible and simple tool to clinically probe the sensory system in uCP. Further research in this field is required to develop quantitative and valid measures of sensory function (e.g., perceptual threshold of touch with electrical stimulation [62] or robotic measures of proprioception [49, 63]) and to link these measures to the underlying mechanisms of the sensory system in uCP.

There are some limitations to be considered for the current study. First, we used scales for the evaluation of lesion location and extent, as well as for assessing sensory function that was based on an ordinal scoring. Although they have been shown to be reliable in uCP [25, 29], such scales may lack sensitivity. Second, our study lacked a neurophysiological technique to probe the sensory system (i.e., sensory evoked potentials) that may contribute to better understand the underlying mechanisms of sensory function in individuals with uCP. Third, the main limitation of the TMS assessment itself lays in the maximum stimulator output intensity that can be reached. This intensity may not have been sufficient to elicit a MEP from either the lesioned or the nonlesioned hemisphere, as the resting motor thresholds are normally higher in children and may be even higher in individuals with uCP. This limitation might have prevented us from finding a CST projection to eventually diagnose the individual as CST_bilat_ or CST_ipsi_ wiring. Furthermore, the MEP data were not analysed, which may provide with useful insights in future studies. Lastly, although our sample size was large and covers the most common lesion timing groups, our results cannot be completely extended to those children with malformations or postnatally acquired brain injuries, as these were not included in the analyses.

## 5. Conclusions

CST wiring mainly determines UL motor function, although also lesion extent and damage to the basal ganglia and thalamus significantly contributed to the prediction of UL motor deficits. For sensory function, lesion extent, timing, and the type of CST wiring pattern seem to be important to develop adequate sensory function. The underlying CST wiring seems to disrupt the association between sensory and motor functions, pointing toward different mechanisms of sensorimotor integration in uCP. The results of our study contribute to a better understanding of the underlying pathophysiology of motor and sensory functions and highlight the importance of investigating sensorimotor integration in future studies. Subsequently, these insights will aid in developing new intervention strategies tailored to the specific deficits of the motor and sensory systems of the individual child with uCP.

## Figures and Tables

**Table 1:** Contingency table (count and percentage, descriptive statistics) of the occurrence of lesion timing, location, and extent according to the CST wiring.

			CST wiring			*p* value
			Contralateral	Bilateral	Ipsilateral	
Timing						
Lesion timing^¥^	PV	*N* (%)	15 (88.2%)	8 (50%)	11 (57.9%)	0.04
	CSC		2 (11.8)	8 (50%)	8 (42.1%)	
Location						
PLIC^¥^	Not affected	*N* (%)	8 (47%)	1 (6%)	0 (0%)	<0.001
	Affected		9 (53%)	15 (94%)	19 (100%)	
Basal ganglia and thalamus^◊^		Me (p25–p75)	0 (0–1)	1.50 (0–2.50)	1 (1–2)	0.006^a,b^
Frontal lobe^◊^		Me (p25–p75)	1 (1–1)	1.50 (1–2.25)	1 (1–1.50)	0.004^a,b^
Parietal lobe^◊^		Me (p25–p75)	2 (1–2)	2 (1.25–3)	2 (2–2.50)	0.09
Extent						
Ipsilesional extent^○^		*X* (SD)	5.18 (3.07)	8.38 (3.95)	9.05 (3.27)	0.004^a,b^

CST: corticospinal tract; PV: periventricular; CSC: corticosubcortical; PLIC: posterior limb of the internal capsule. ^¥^Chi-square statistic. ^§^Fisher's exact test. ^◊^Kruskal-Wallis test. ^○^ANOVA. ^a^Contralateral vs. ipsilateral. ^b^Contralateral vs. bilateral.

**Table 2:** Descriptive statistics of the observed and estimated marginal means of upper limb motor function according to the CST wiring groups.

	Estimated marginal means and SD		
	CST_contra_ (*n* = 16)	CST_ipsi_ (*n* = 19)	CST_bilat_ (*n* = 16)
Grip strength ratio (log)^a^	0.14 (0.13)	0.55 (0.20)	0.46 (0.24)
JTHFT ratio (log)^b^	0.30 (0.24)	0.67 (0.23)	0.64 (0.22)
AHA (0–100)^c^	79.66 (10.28)	58.70 (9.81)	61.58 (9.67)

CST: corticospinal tract; JTHFT: Jebsen-Taylor hand function test; AHA: Assisting Hand Assessment; SD: standard deviation. ^a^The values coincide with the observed values, as there is no significant covariate in the model. ^b^Adjustments based on ipsilesional lesion extent mean = 7.67. ^c^Adjustments based on ipsilesional lesion extent mean = 7.67 and damage to the basal ganglia and thalamus mean = 1.12.

**(a):** 

		Stereognosis (number of correctly guessed objects)						
		0	1	2	3	4	5	6
Lesion timing								
PV	*N* (%)	0 (0%)	0 (0%)	1 (25%)	0 (0%)	5 (71%)	6 (67%)	17 (44%)
CSC	*N* (%)	5 (100%)	2 (100%)	3 (75%)	1 (100%)	2 (29%)	3 (33%)	1 (6%)
CST wiring								
Contralateral	*N* (%)	0 (0%)	0 (0%)	1 (25%)	0 (0%)	0 (0%)	1 (11%)	13 (72%)
Bilateral	*N* (%)	4 (80%)	0 (0%)	2 (50%)	0 (0%)	3 (43%)	3 (33%)	3 (17%)
Ipsilateral	*N* (%)	1 (20%)	2 (100%)	1 (25%)	1 (100%)	4 (57%)	5 (56%)	2 (11%)
Lesion extent								
Ipsilesional	Me (IQR)	13 (2.07)	13 (—)	10 (3.88)	—	6 (3.50)	6 (5.25)	5.25 (3.75)

**(b):** 

		Two-point discrimination	
		Normal (≤4 mm)	Impaired (>5 mm)
Lesion timing			
PV	*N* (%)	26 (93%)	3 (17%)
CSC	*N* (%)	2 (7%)	15 (83%)
Lesion extent			
Ipsilesional	Me (IQR)	5.25 (3.88)	12 (5.25)

**(c):** 

		Threshold of touch sensation				
		Normal	Diminished light touch	Diminished protective sensation	Loss of protective sensation	Untestable
Lesion extent						
Ipsilesional	Me (IQR)	6 (4.50)	—	10.50 (11.25)	13 (2.41)	12.50 (—)

PV: periventricular lesion; CSC: corticosubcortical lesion; CST: corticospinal tract; *N*: number of cases; Me: median; IQR: interquartile range.

## Data Availability

All data concerning this study is available within the manuscript. Detailed data is available upon request to the first author.

## Supplementary Materials

Supplementary Materials Table 1: descriptive information of the distribution of the lesion location and extent according to the lesion timing groups. Table 2: descriptive statistics (*X* (SD)) and univariate analysis of upper limb motor function according to the CST wiring and the brain lesion characteristics. Table 3: descriptive statistics (Me (IQR)) and univariate analysis of upper limb sensory function (3A, stereognosis and 3B, two-point discrimination and thresholds of touch sensation) according to the CST wiring and the brain lesion characteristics.
