# Supplementary material for: Corticospinal Tract Wiring and Brain Lesion Characteristics in Unilateral Cerebral Palsy: Determinants of Upper Limb Motor and Sensory Function
Source: Neural Plast. 2018 Sep 13;2018:2671613. doi: 10.1155/2018/2671613 (PMC6158964; doi:10.1155/2018/2671613)
Supplement: Supplementary Materials — Table 1: descriptive information of the distribution of the lesion location and extent according to the lesion timing groups. Table 2: descriptive statistics (X (SD)) and univariate analysis of upper limb motor function according to the CST wiring and the brain lesion characteristics. Table 3: descriptive statistics (Me (IQR)) and univariate analysis of upper limb sensory function (3A, stereognosis and 3B, two-point discrimination and thresholds of touch sensation) according to the CST wiring and the brain lesion characteristics. [file 2671613.f1.pdf]

# Supplementary materials

# Corticospinal tract wiring and brain lesion characteristics in unilateral cerebral palsy: determinants of upper limb motor and sensory function

Cristina Simon-Martinez, Ellen Jaspers, Lisa Mailleux, Els Ortibus, Katrijn Klingels, Nicole Wenderoth, and Hilde Feys

Table 1. Descriptive information of the distribution of the lesion location and extent according to the lesion timing groups.

| | | | PV lesions | CSC lesions |
|---|---|---|---|---|
| **PLIC** | **Affected** | N (%) | 26 (61%) | 17 (40%) |
| | **Not affected** | N (%) | 8 (89%) | 1 (11%) |
| **Frontal lobe** | | Me (IQR) | 1 (0) | 1.50 (1.13) |
| **Parietal lobe** | | Me (IQR) | 1.75 (1) | 3 (0.63) |
| **Basal ganglia and thalamus** | | Me (IQR) | 0 (1) | 2 (4.63) |
| **Lesion extent** | | Me (IQR) | 5.75 (4) | 12 (2) |

PLIC, posterior limb of the internal capsule; PV, periventricular; CSC, cortico-subcortical.

Table 2. Descriptive statistics (X (SD)) and univariate analysis of upper limb motor function according to the CST wiring and the brain lesion characteristics.

| | | Grip strength | | | JTHFT | | | AHA | |
|---|---|---|---|---|---|---|---|---|---|
| | | $R^2$ (p-value) | Aff. Hand (Kg) | Ratio (Non-aff/Aff) | $R^2$ (p-value) | Aff. Hand (s) | Ratio (Non-aff/Aff) | $R^2$ (p-value) | Log units |
| **CST wiring** | | 0.47 (<0.0001) | | | 0.46 (<0.0001) | | | 0.52 (<0.0001) | |
| **Contralateral** | | | 14.45 (6.84) | 1.45 (0.53) | | 71.72 (55.89) | 1.89 (1.13) | | 81.31 (8.57) |
| **Bilateral** | | | 7.57 (8.54) | 3.27 (1.67) | | 196.25 (142.66) | 5.30 (2.82) | | 61.63 (11.06) |
| **Ipsilateral** | | | 6.16 (4.51) | 3.97 (2.01) | | 239.29 (169.78) | 5.99 (3.29) | | 57.26 (11.07) |
| **Timing** | | 0.14 (0.008) | | | 0.23 (<0.001) | | | 0.14 (0.008) | |
| **PV** | | | 11.32 (8.23) | 2.50 (1.74) | | 115.55 (89.32) | 3.35 (2.39) | | 70.09 (13.34) |
| **CSC** | | | 5.32 (3.72) | 3.80 (1.87) | | 278.94 (181.65) | 6.56 (3.36) | | 59.00 (14.40) |
| **Location** | | | | | | | | | |
| **PLIC** | **Not affected** | 0.19 (0.001) | 16.52 (6.76) | 1.37 (0.22) | 0.16 (0.004) | 65.63 (21.16) | 1.86 (0.51) | 0.20 (0.001) | 81.00 (7.25) |
| | **Affected** | | 7.84 (6.88) | 3.25 (1.90) | | 193.23 (155.40) | 4.97 (3.19) | | 62.81 (13.55) |
| **Basal ganglia and thalamus** | | 0.09 (0.03) | | | 0.16 (0.004) | | | 0.12 (0.01) | |
| **Frontal lobe** | | 0.08 (0.04) | | | 0.23 (<0.001) | | | 0.16 (0.003) | |
| **Parietal lobe** | | 0.15 (0.005) | | | 0.15 (0.006) | | | 0.12 (0.01) | |
| **Ipsilesional extent** | | 0.20 (<0.001) | | | 0.30 (<0.0001) | | | 0.27 (<0.0001) | |

CST, corticospinal tract; PV, periventricular lesión; CSC, cortico-subcortical; PLIC, posterior limb of the internal capsule; JTHFT, Jebsen-Taylor Hand Function Test; AHA, Assisting Hand Assessment; Kg, kilograms; s, seconds; Aff, affected; Non-aff, non-affected; $R^2$, R-squared value from the regression model; p-value, probability value based on the regression model.

Table 3. Descriptive statistics (Me (IQR)) and univariate analysis of upper limb sensory function (3A, stereognosis and 3B, two-point discrimination and thresholds of touch sensation) according to the CST wiring and the brain lesion characteristics.

**3A. Stereognosis**

| | | | Stereognosis (number of objects correctly recognized) | | | | | | | |
|---|---|---|---|---|---|---|---|---|---|---|
| | | | **Wald $Chi^2$ (p-value)** | **0** | **1** | **2** | **3** | **4** | **5** | **6** |
| **CST wiring** | | N (%) | 14.97 (0.001) | | | | | | | |
| **Contralateral** | | | | 0 (0%) | 0 (0%) | 1 (25%) | 0 (0%) | 0 (0%) | 1 (11%) | 13 (72%) |
| **Bilateral** | | | | 4 (80%) | 0 (0%) | 2 (50%) | 0 (0%) | 3 (43%) | 3 (33%) | 3 (17%) |
| **Ipsilateral** | | | | 2 (20%) | 2 (100%) | 1 (25%) | 1 (100%) | 4 (57%) | 5 (56%) | 2 (11%) |
| **Timing** | | N (%) | 19.05 (<0.0001) | | | | | | | |
| **PV** | | | | 0 (0%) | 0 (0%) | 1 (25%) | 0 (0%) | 5 (71%) | 6 (67%) | 17 (94%) |
| **CSC** | | | | 5 (100%) | 2 (100%) | 3 (75%) | 1 (100%) | 2 (29%) | 3 (33%) | 1 (6%) |
| **Location** | | N (%) | | | | | | | | |
| **PLIC** | **Not affected** | | 4.57 (0.03) | 0 (0%) | 0 (0%) | 0 (0%) | 0 (0%) | 0 (0%) | 2 (22%) | 5 (28%) |
| | **Affected** | | | 5 (100%) | 2 (100%) | 4 (100%) | 1 (100%) | 7 (100%) | 7 (78%) | 13 (72%) |
| **Basal ganglia and thalamus** | | Me (IQR) | 20.28 (<0.0001) | 3 (1) | 2 (0) | 1.5 (1.75) | 3 (-) | 1 (1) | 0 (2) | 0 (1) |
| **Frontal lobe** | | Me (IQR) | 17.88 (<0.0001) | 2.50 (1) | 2.25 (-) | 1.50 (1.50) | 3 (-) | 1 (0.50) | 1 (0.63) | 1 (0.13) |
| **Parietal lobe** | | Me (IQR) | 13.78 (<0.001) | 3 (0.25) | 3 (0) | 2.75 (1.25) | 2 (-) | 2 (1.50) | 2 (1.38) | 1.75 (2) |
| **Ipsilesional extent** | | Me (IQR) | 21.20 (<0.0001) | 13 (2.07) | 13 (-) | 10 (3.88) | 15.50 (-) | 6 (3.50) | 7 (4.25) | 5.25 (3.75) |

Table 3 (continuation).

**3B. Two-point discrimination and touch sensation**

| | | | Two-point discrimination | | | Thresholds of touch sensation | | | | | |
|---|---|---|---|---|---|---|---|---|---|---|---|
| | | | Wald $Chi^2$ (p-value) | Normal | Impaired | Wald $Chi^2$ (p-value) | Normal | Diminished light touch | Diminished protective sensation | Loss of protective sensation | Untestable |
| **CST wiring** | | N (%) | 6.71 (0.04) | | | 1.74 (0.42) | | | | | |
| **Contralateral** | | | | 14 (50%) | 1 (6%) | | 6 (26%) | 0 (0%) | 1 (25%) | 0 (0%) | 0 (0%) |
| **Bilateral** | | | | 7 (25%) | 8 (44%) | | 9 (39%) | 0 (0%) | 0 (0%) | 3 (50%) | 2 (100%) |
| **Ipsilateral** | | | | 7 (25%) | 9 (50%) | | 8 (34%) | 0 (0%) | 7 (75%) | 3 (50%) | 0 (0%) |
| **Timing** | | N (%) | 18.57 (<0.0001) | | | 10.64 (0.001) | | | | | |
| **PV** | | | | 26 (93%) | 3 (17%) | | 18 (78%) | 0 (0%) | 1 (25%) | 0 (0%) | 0 (0%) |
| **CSC** | | | | 2 (7%) | 15 (83%) | | 5 (22%) | 0 (0%) | 3 (75%) | 6 (100%) | 2 (100%) |
| **Location** | | N (%) | | | | | | | | | |
| **PLIC** | **Not affected** | | 1.85 (0.17) | 6 (21%) | 1 (6%) | 0.00 (0.99) | 2 (9%) | 0 (0%) | 0 (0%) | 0 (0%) | 0 (0%) |
| | **Affected** | | | 22 (79%) | 17 (94%) | | 21 (91%) | 0 (0%) | 4 (100% | 6 (100%) | 2 (100%) |
| **Basal ganglia and thalamus** | | Me (IQR) | 11.08 (0.001) | 0.50 (1) | 2 (2) | 9.97 (0.002) | 1 (2) | - | 1.50 (2.50) | 2.50 (1.25) | 2.50 (-) |
| **Frontal lobe** | | Me (IQR) | 9.41 (0.002) | 1 (0) | 1.50 (1.25) | 12.83 (<0.001) | 1 (0) | - | 1.50 (1.88) | 2.75 (1.50) | 2.25 (-) |
| **Parietal lobe** | | Me (IQR) | 10.80 (0.001) | 1.50 (1.13) | 3 (0.75) | 8.55 (0.003) | 1.5 (1) | - | 2.50 (2.50) | 3 (0.13) | 3 (0) |
| **Ipsilesional extent** | | Me (IQR) | 12.74 (<0.0001) | 5.25 (4) | 12.50 (4.75) | 10.75 (0.001) | 6 (4.50) | - | 10.50 (11.25) | 13 (2.41) | 12.50 (-) |

CST, corticospinal tract; PV, periventricular lesion; CSC, cortico-subcortical; PLIC, posterior limb of the internal capsule; Aff, affected; Non-aff, non-affected; p-value, probability value based on the regression model.
